# Clinical utility of plasma miR‐371a‐3p in germ cell tumors

**DOI:** 10.1111/jcmm.14013

**Published:** 2018-12-07

**Authors:** Michal Mego, Ton van Agthoven, Paulina Gronesova, Michal Chovanec, Vera Miskovska, Jozef Mardiak, Leendert H. J. Looijenga

**Affiliations:** ^1^ Translational Research Unit Faculty of Medicine Comenius University and National Cancer Institute Bratislava Slovakia; ^2^ 2nd Department of Oncology Faculty of Medicine Comenius University and National Cancer Institute Bratislava Slovakia; ^3^ Department of Pathology Laboratory for Experimental Patho‐Oncology Erasmus MC University Medical Center Cancer Institute Rotterdam The Netherlands; ^4^ Cancer Research Institute Biomedical Research Center Slovak Academy of Sciences Bratislava Slovakia; ^5^ 1st Department of Oncology Faculty of Medicine Comenius University and St. Elisabeth Cancer Institute Bratislava Slovakia; ^6^ Princess Maxima Center Pediatric Oncology Utrecht The Netherlands; ^7^ Present address: Prinses Máxima Centrum for Pediatric Oncology Utrecht The Netherlands

**Keywords:** miR‐371a‐3p, prognostic liquid biopsy biomarker, (testicular) germ cell tumours

## Abstract

Germ cell tumours predominantly of the testis ((T)GCTs) are remarkably chemotherapy sensitive. However, a small proportion of patients fail to be cured with cisplatin‐based combination chemotherapy. miR‐371a‐3p is a new liquid biopsy biomarker for (T)GCTs. The aim of this study was to evaluate clinical utility of plasma miR‐371a‐3p level in patients starting systemic chemotherapy. Patients were included before the first cycle (N = 180) and second cycle (N = 101) of systemic first line chemotherapy, treated between July 2010 and May 2017. Plasma miR‐371a‐3p levels were measured with the ampTSmiR test and compared to disease characteristics and outcome. Pretreatment plasma miR‐371a‐3p levels were increased in 51.7% of cases and associated with number of metastatic sites, presence of lung, retroperitoneal, and mediastinal lymph node metastases, S – stage, IGCCCG risk group, and response to therapy. Patients with a negative pretreatment plasma level had better progression‐free survival (PFS) and overall survival (OS) compared to patients being positive for miR‐371a‐3p (hazard ratio [HR] = 0.26, 95% confidence interval [CI] 0.09‐0.71, *P *=* *0.02 for PFS and HR = 0.21, 95% CI 0.07‐0.67, *P *=* *0.03 for OS, respectively). Patients negative for miR‐371a‐3p in both samples had a superior PFS (HR = 0.10, 95% CI 0.01‐21.49, *P* = 0.02) and OS (HR = 0.08, 95% CI 0.01‐27.81, *P* = 0.008) compared to patients with miR‐371a‐3p positive in both samples (multivariate analyses were non‐significant). In total 68% of the patients were S0. This study demonstrates clinical value of plasma miR‐371a‐3p level in chemotherapy naïve (T)GCT patients starting first line of chemotherapy to predict prognosis.

## 1 INTRODUCTION

Testicular germ cell tumours (TGCTs) are rare, although they are the most common cancer in young Caucasian males, accounting for 60% of all malignancies in the age group between 20 and 40 years.^1^, ^2^ TGCTs represent a highly curable malignancy. In fact, cisplatin based chemotherapy is the mainstay in the treatment of TGCTs, resulting in about 70%‐80% cure of patients with disseminated disease with a multimodality approach. However, between 20% and 30% of patients do not respond or relapse with this treatment protocol.^2^, ^3^ About 20%‐25% of patients with relapsed TGCTs may be cured with various approaches, including salvage chemotherapy based on high‐dose chemotherapy with autologous hematopoietic stem‐cell rescue or combined with standard cisplatin dose and previously non‐utilized drugs.^4^, ^5^, ^6^

Currently, the clinical management of advanced TGCT disease is based on classical serum protein markers (alpha‐fetoprotein [AFP], β‐human chorionic gonadotropin [HCG], and lactate dehydrogenase [LDH]), and imaging studies.^7^, ^8^ However, only 60% of TGCTs demonstrate elevated serum tumour markers at initial diagnosis. Therefore, in advanced cases, there is a need to identify reliable markers capable of determining chemotherapy effectiveness and patients’ outcome. microRNAs (miRNAs) are involved in important biological processes, both normal as well as pathological, including various types of cancer. As the discovery of specific embryonic stem cell related miRNAs as relevant oncogenes in TGCTs,^9^ various studies evaluated their role in tissues and body fluids of (T)GCT patients.^9^, ^10^, ^11^, ^12^, ^13^, ^14^, ^15^, ^16^, ^17^

In the largest series to date of 250 TGCT patients, 60 non‐TGCT patients at primary diagnosis and 104 male healthy donors, the detection of miR‐371a‐3p, 373‐3p, and 367‐3p in sera with the ampTSmiR test was highly informative to diagnose patients with the primary TGCTs. The data showed an area under the curve (AUC) of 0.96, with a 90% sensitivity and 91% specificity.^14^ In an independent study performed with a slightly different protocol and consisting of a series of sera from 166 TGCTs patients and 118 male controls, a positive miR‐371a‐3p level was found in 88.7% of the patients with the AUC of 0.94, and a 93.4% specificity.^10^ Both studies demonstrated that the detection of miR‐371a‐3p in serum is far more sensitive and specific than the classical serum markers β‐HCG, AFP, and LDH. Furthermore, the miR‐371a‐3p levels correlate with tumour burden and treatment results.^10^ In addition, the four‐serum embryonic stem cell related miRNA panel (including miR‐371a‐3p, miR‐372‐3p, miR‐373‐3p, and miR‐367‐3p) also showed a high sensitivity/specificity for diagnosing pediatric (mainly extra‐gonadal) GCTs, based on serum and cerebrospinal fluid analyses. It allowed early detection of relapse of a single mixed tumour and distinguished intracranial malignant GCTs from intracranial non‐GCTs at the time of diagnosis.^15^ In addition, the ampTSmiR test targeting miR‐371a‐3p used in our laboratory is more sensitive than the conventional TGCTs protein serum biomarkers for the detection of residual disease and relapse.^13^, ^14^, ^18^ In conclusion, miR‐371a‐3p is the most promising miRNA for the detection of (T)GCTs in body fluids to date, performed with a highly sensitive and specific multiplexed pre‐amplification quantitative real‐time PCR (qRT‐PCR) technique.^10^, ^14^

However, in spite of the fact that miR‐371a‐3p is a promising new specific biomarker for (T)GCTs, data related to its clinical utility are still limited.^9^, ^10^, ^11^, ^12^, ^18^ The aim of this study was to evaluate clinical utility of plasma miR‐371a‐3p level in chemo‐naïve GCTs patients starting systemic chemotherapy.

## 2 MATERIALS AND METHODS

### 2.1 Study patients

This retrospective translational study (Protocol IZLO1, Chair: M. Mego) was approved by the Institutional Review Board (IRB) of the National Cancer Institute and was conducted between July 2010 and May 2017. All consecutive patients with TGCTs treated with orchiectomy (except two patients that started treatment without orchiectomy due to very advanced disease) and at least one cycle of cisplatin‐based chemotherapy in the National Cancer Institute of Slovakia and St. Elisabeth Cancer Institute in Slovakia were enrolled in this prospective study. Data regarding age, tumour histological subtype, clinical stage, type, and number of sites of metastasis and type of chemotherapy regimen were recorded in all the patients and compared with plasma miR‐371a‐3p level (Table 1). TGCT patients were recruited and consented according to the Institutional Review Board approved protocol. The study adhered to the “Code for Proper Secondary Use of Human Tissue in The Netherlands” developed by the Dutch Federation of Medical Scientific Societies (FMWV) (Version 2002, update 2011). STARD guidelines were followed to perform this study.^19^

**Table 1:** Patients’ characteristics

Variable	N	%
All patients	180	100.0
Histology		
Seminoma	51	28.3
Non‐seminoma	127	70.6
Unknown^a^	2	1.1
Primary tumour localization		
Testicular	169	93.9
Extragonadal	11	6.1
IGCCCG risk group	105	58.3
Good risk	21	11.7
Intermediate risk	22	12.2
Poor risk	32	17.8
Sites of metastases	45	25.0
Retroperitoneum	109	60.6
Mediastinum	26	14.4
Lungs	–	–
Liver	57	31.7
Brain	123	68.3
Other	–	–
Visceral non‐pulmonary metastases	159	88.3
No. of metastatic site		
0	21	11.7
1	144	80.0
2	36	20.0
>3	–	–
Staging (UICC)		
IA	5	2.8
IB	27	15.0
IS	13	7.2
IIA	19	10.6
IIB	26	14.4
IIC	16	8.9
IIIA	26	14.4
IIIB	21	11.7
IIIC	27	15.0
Response to therapy		
Favourable response	173	96.1
Unfavourable response	7	3.9
Plasma samples		
Samples before first cycle of chemotherapy	180	100.0
Samples before second cycle of chemotherapy	101	56.1
No. of positive samples		
Before first cycle of chemotherapy	93	51.7
Before second cycle of chemotherapy	4	2.2
Median value of miR‐371a‐3p		
Before first cycle of chemotherapy (range)	1.2	0‐477.4
Before second cycle of chemotherapy (range)	0.0	0‐8.6

IGCCCG, International Germ Cell Consensus Classification Group; UICC, Union for International Cancer Control.

a Diagnosis based on typical clinical presentation and highly elevated serum tumour markers.

### 2.2 Samples collection

Peripheral blood samples were collected from all translational study participants into BD Vacutainer^®^ EDTA tubes at baseline in the morning on day −1 or 0 of the first cycle of chemotherapy (n = 180) and before the second cycle of chemotherapy (n = 101). Patients’ blood samples (5 mL) were centrifuged at 2300 *g* for 10 minutes to separate the plasma and blood cells. Collected plasma samples were afterwards filtered through a 0.2 μm filter to remove larger particles. Plasma aliquots were stored at −80°C until further analysis. In total, 281 plasma samples of 180 patients were analysed (Table 1). Median age of patients was 30 years (range 16‐67 years).

The miRNA levels of (T)GCT patients were compared with plasma levels of 50 male healthy donors, of which also serum was available taken at the same time point. Plasma was collected using Vacuette K3 EDTA Liquid, 6 ml vials (Greiner Bio‐One, Alphen aan den Rijn, the Netherlands).

The median age was 54 (range 22‐70 years). In our previously reported study, we showed that age did not influence the outcome of the miRNA analysis in sera^14^ (additional samples Person coefficient of 0.2336, *P *= 0.26), nor did it influence the outcome in this series of plasma samples. Because of the specific regulations it is not allowed to have information related to clinical data of the control group, except age, and gender. The donors of Sanquin are supposedly healthy males, but not specifically checked for absence of a T(GCT). However, based on the incidence in the Netherlands being around 800 patients per year in a population of ~8 million males, the chance of including a patient is very low. It was not possible to collect large numbers of control plasmas in Slovenia. However, both populations were from Caucasian origin. At present no reports were made according to population differences and expression of miR‐371 in T(GCT). The normal control plasma samples were obtained from Sanquin (Amsterdam, the Netherlands).

### 2.3 miRNA purification and RT‐qPCR

For the ampTSmiR tests specific miRNAs were isolated from 50 μL plasma using target‐specific anti‐miRNA magnetic beads as described before.^14^ In short, cDNA generation and quantification of miRNA levels were performed with a highly sensitive multiplexed pre‐amplification quantitative RT‐PCR technique (ampTSmiR test). The following TaqMan MicroRNA assays were used: catalog ID: hsa‐miR‐371a‐3p (002124); ath‐miR‐159a (000338), and hsa‐miR‐30b‐5p (000602) (Thermo Fisher Scientific, Bleiswijk, the Netherlands): In brief 5 μL of specifically targeted purified miRNA in elution buffer was reverse transcribed into miRNA‐specific cDNA with a TAQMAN(R) MICRORNA RT KIT (Thermo Fisher Scientific), followed by a 13‐cycle pre‐amplification step using 2x TaqMan Preamp Master mix (4488593, detailed protocol by supplier Thermo Fisher Scientific) and 20x TaqMan MicroRNA Assays. Thermal‐cycling conditions: 95°C for 10 minutes, followed by 13 cycles of 95°C for 15 seconds and 60°C for 1 minute. miRNA levels were determined in 1.5 μL of cDNA on a TaqMan 7500 Real‐Time PCR system, according to the supplier (Thermo Fisher Scientific).

### 2.4 Quality control

A non‐human miRNA spike‐in ath‐miR‐159a was added in the same fixed amount to the sera (0.2 μL of a 1 nmol/L stock solution) for quality control of RNA isolation and cDNA generation. For calibration of input miRNA levels ath‐miR‐159a was used. All plasma samples were visually inspected and no hemolytic samples were present that could lead to false interpretation of the results. No samples had to be excluded due to poor miRNA recovery, based on recovery of the spike‐in ath‐mir‐159a (variation in Ct generally within ± 2 Cts, n = 301, SD < 1.67). For normalization the mean levels of the endogenous reference miRNA (miR‐30b‐5p) were used as described before.^14^, ^15^ In each cDNA synthesis experiment, five 10‐fold dilution series of purified miRNA of the TCam‐2 seminoma cell line was included for quality control and qPCR efficiency and interplate calibration. For negative control, the no template control, elution buffer was added instead of purified miRNA as described previously.^14^

### 2.5 Evaluation

The target miRNA level per sample was determined according to 2−ΔΔCT method.^15^ Threshold for miR‐371a‐3p was calculated using plasma samples of healthy donors analysed in this study (Figure S1). A cut‐off value of ≥2.0 (Ct = 29.26) of the highest level (Ct = 30.26) observed in the healthy donor group was used to dichotomize the samples in two categories as positive or negative (cut‐off for miR‐371a‐3p = 1) as described earlier.^15^ Plasma samples of healthy donors (n = 50) contained similar minimal levels of miR‐371a‐3p as matched sera, and control sera used in a previous study (n = 104)^14^ (Figure S1).

### 2.6 Statistical analysis

Patient data were tabulated. The patients’ characteristics were summarized using the mean or median (range) for continuous variables and frequency (percentage) for categorical variables, respectively. Statistical analysis was performed using non‐parametric tests as the distribution of the miR‐371a‐3p levels was significantly different from the normal distribution (Shapiro‐Wilk test). The Mann‐Whitney *U* test was used for the analysis of the association of the miR‐371a‐3p expression to clinicopathological variables between the two groups of patients, and Kruskal‐Wallis test among more than two groups, whereas Fisher's exact test or the χ^2^ test were used when miR‐371a‐3p expression was categorized as positive or negative. Wilcoxon test was used to compare plasma the miR‐371a‐3p before the first and second cycle of chemotherapy. Pearson's correlations were used to determine correlation between serum tumour markers and plasma miR‐371a‐3p.

Median follow‐up period was calculated from the date of the starting treatment with cisplatin‐based chemotherapy, as a median observation time among all patients and among those still alive at the time of their last follow‐up. Progression‐free survival (PFS) was calculated from the date of the starting treatment with cisplatin‐based chemotherapy to the date of progression or death or the date of the last adequate follow‐up. Overall survival (OS) was calculated from the date of starting treatment with cisplatin‐based chemotherapy to the date of death or last follow‐up. Survival rates were estimated using the Kaplan‐Meier product limits method and were compared with the log‐rank test to determine significance. miR‐371a‐3p expression data were dichotomized into positive and negative based on the miR‐371a‐3p plasma level (see above). A multivariate Cox proportional hazards model for PFS and OS was used to assess differences in outcome on the basis of the miR‐371a‐3p expression in primary tumour and/or biopsy of metastatic site and prognosis according to the IGCCCG (International Germ Cell Collaborative Group) criteria (IGCCCG, 1997). The Pearson correlation coefficient was used to examine a correlation between miR‐371a‐3p plasma levels and S‐stage (as defined by IGCCCG criteria; S0 within normal limits; S1, AFP < 1000 ng/mL and/or β‐HCG < 5000 mIU/mL and/or LDH < 1.5 U/L upper normal limit; S2, AFP 1000‐10 000 ng/mL and/or β‐HCG 5000‐50 000 mIU/mL and/or LDH 1.5‐10 U/L upper normal limit; S3 AFP > 10 000 ng/mL and/or β‐HCG > 50 000 mIU/mL and/or LDH > 10 U/L upper normal limit).

Data processing and statistical analysis were performed using Microsoft Excel 2010, GraphPad Prism 7 (GraphPad Software, La Jolla, CA, USA) and NCSS software (NCSS, LLC, East Kaysville, Utah, USA). A *P* < 0.05 was considered statistically significant.

## 3 RESULTS

### 3.1 Patient characteristics

From July 2010 and May 2017, 180 patients starting adjuvant and/or new line of chemotherapy were registered to the study. Basic characteristic of the patients is shown in Table 1. The majority of patients had primary TGCT (93.9%) and non‐seminoma histology (70.6%). The study population included 32 patients with stage I disease (five patients stage IA and 27 patients stage IB) treated with adjuvant chemotherapy. All these patients had non‐seminoma histology, in 27 of tumours (84.4%) lymph vascular invasion was present, while, 29 of them exhibit (90.6%) embryonal carcinoma component. No relapse and/or death was observed in patients with stage I disease.

All patients were treated with platinum‐based chemotherapy, the majority received BEP (bleomycin, etoposide, cisplatin) regimen 142 (78.9%), 23 patients (12.8%) received EP (etoposide, cisplatin) chemotherapy, 6 (3.3%) received TIP (paclitaxel, ifosfamide, cisplatin) chemotherapy, five patients (2.8%) VIP (etoposide, ifosfamide, cisplatin), and four patients (2.2%) were treated with dose‐dense chemotherapy.^19^

### 3.2 Association between plasma miR‐371a‐3p level and patients/tumour characteristics

Analysis between plasma miR‐371a‐3p level and patients/tumour characteristics included all 180 patients. Pretreatment plasma miR‐371a‐3p levels were significantly associated with IGCCCG risk group, number of metastatic sites, presence of retroperitoneal and mediastinal lymph node metastases, lung metastases, S – stage, and favourable response to therapy (Table 2). These associations were consistent for plasma miR‐371a‐3p as continuous variable or dichotomized as positive vs negative, except for response to therapy. Subgroup analysis showed that both associations were triggered mainly by non‐seminomas (Tables S1 and S1), where plasma miR‐371a‐3p was associated with primary localization of the tumour as well. In S0 stage patients that represented 73 (40.6%) of study population, there were no associations between plasma miR‐371a‐3p and disease characteristics (Table S3). None of the five patients with stage IA were miR‐371a‐3p positive, while four out of 27 patients (29.6%) with stage IB were miR‐371a‐3p positive and two out of 13 (15.4%) of patients with IS stage were miR‐371a‐3p positive. Overall, six of 45 patients (13.3%) with CSI were miR‐371a‐3p positive.

**Table 2:** Association between miR‐371a‐3p and patients/tumour characteristics

Variable	N	Mean	SEM	Median	*P*‐value	Negative		Positive		*P*‐value
						N	%	N	%	
All patients	180	25.5	1.2	4.9	NA	87	48.3	93	51.7	NA
Histology										
Seminoma	51	36.6	3.4	9.2	0.23	19	37.3	32	62.7	**0.07**
Non‐seminoma	127	21.1	0.8	5.8		68	53.5	59	46.5	
Unknown histology	2									
Tumour primary										
Primary TGCTs	169	25.2	1.1	5.0	0.46	82	48.5	87	51.5	1.00
Extragonadal GCTs	11	30.7	7.9	19.7		5	45.5	6	54.5	
IGCCCG risk group										
Good risk	105	18.7	1.6	6.1	**<0.00001**	48	45.7	57	54.3	**<0.00001**
Intermediate risk	21	57.0	22.7	13.6		6	28.6	15	71.4	
Poor risk	22	63.2	14.7	13.3		5	22.7	17	77.3	
Stage I (adjuvant therapy)	32	1.2	0.0	11.0		28	87.5	4	12.5	
Number of metastatic sites										
0	45	1.1	0.0	9.1	**<0.00001**	39	86.7	6	13.3	**<0.00001**
1‐2	109	22.3	3.3	5.8		42	38.5	67	61.5	
>3	26	81.0	28.1	11.9		6	23.1	20	76.9	
Retroperitoneal LN metastases										
Absent	57	9.0	0.0	8.5	**<0.00001**	47	82.5	10	17.5	**<0.00001**
Present	123	33.1	5.5	5.8		40	32.5	83	67.5	
Mediastinal lymph nodes metastases										
Absent	159	16.2	0.9	4.8	**0.00010**	82	51.6	77	48.4	**0.02**
Present	21	95.9	28.5	13.1		5	23.8	16	76.2	
Lung metastases										
Absent	144	18.5	0.4	5.3	**0.00001**	79	54.9	65	45.1	**0.001**
Present	36	53.6	23.6	10.7		8	22.2	28	77.8	
Liver metastases										
Absent	169	23.2	1.0	5.0	0.18	84	49.7	85	50.3	0.21
Present	11	60.1	12.6	19.6		3	27.3	8	72.7	
Non‐pulmonary visceral metastases										
Absent	166	23.7	1.1	5.1	0.48	81	48.8	85	51.2	0.78
Present	14	47.3	5.6	17.4		6	42.9	8	57.1	
S – stage^a^										
0	73	2.3	0.0	7.0	**<0.00001**	53	72.6	20	27.4	**<0.00001**
1	61	20.0	2.1	7.6		25	41.0	36	59.0	
2	27	61.2	22.7	11.5		6	22.2	21	77.8	
3	19	81.4	25.9	13.7		3	15.8	16	84.2	
Response to therapy										
Favourable response	173	23.7	1.0	4.9	**0.02**	86	49.7	87	50.3	0.12
Unfavourable response	7	69.9	12.6	24.5		1	14.3	6	85.7	

Statistically significant indicated bold.

TGCTs, testicular germ cell tumours; IGCCCG, International Germ Cell Consensus Classification Group*defined by IGCCCG criteria; S0, within normal limits; S1, AFP < 1000 ng/mL and/or β‐HCG < 5000 mIU/mL and/or LDH < 1.5 U/L upper normal limit; S2, AFP 1000‐10 000 ng/mL and/or β‐HCG 5000‐50 000 mIU/mL and/or LDH 1.5‐10 U/L upper normal limit; S3, AFP > 10 000 ng/mL and/or β‐HCG > 50 000 mIU/mL and/or LDH > 10 U/L upper normal limit; SEM, standard error of the mean.

a Cut‐off for miR‐371a‐3p = 6.89, based on optimal separation of healthy donors and primary (T)GCT patients.

The associations between the histological subtypes of the primary (T)GCT and the plasma miR‐371a‐3p levels are summarized in Table S4. Within the whole group, there was no association between histology of primary tumour and plasma miR‐371a‐3p levels. In stage I disease, there were no differences in plasma miR‐371a‐3p levels based on histology of primary tumour.

**Figure 1:**
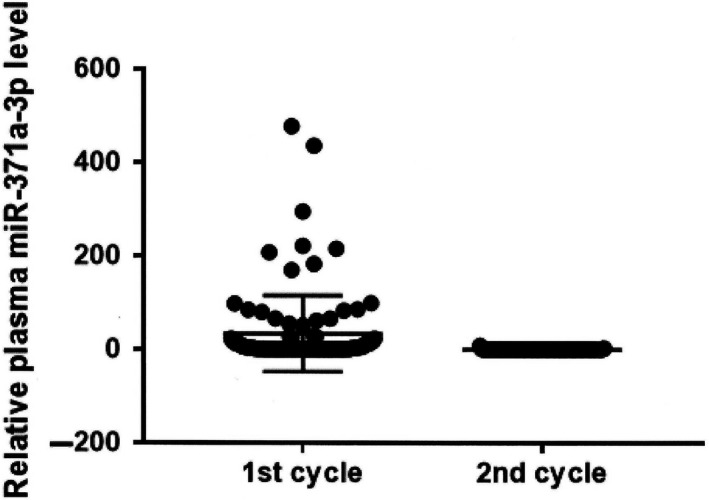
Relative miR‐371a‐3p plasma level before the first and second cycle of chemotherapy (hazard ratio [HR] = 0.26, 95% confidence interval [CI] 0.09‐0.71, *P* = 0.02 for PFS and HR = 0.21, 95% CI 0.07‐0.67, *P* = 0.03 for OS, respectively) (Table 4, Figure 2A,B)

Analysis of 101 paired samples (corresponding samples before starting of first cycle and second cycle of chemotherapy) showed that mean plasma level of miR‐371a‐3p was significantly higher before the first cycle compared to the second cycle of chemotherapy (mean ± SEM = 33.6 ± 8.1 vs. 0.2 ± 0.1, *P *<* *0.00001) (Figure 1). Of 53 patients (52.5%) that were positive for pretreatment miR‐371a‐3p, only two patients (3.8%), remained positive, one with significant (from 99.9 to 1.5) and second with minor decrease (from 3.3 to 2.2) of plasma miR‐371a‐3p, while two patients (2.0%), initially miR‐371a‐3p negative, became positive before the second cycle of chemotherapy (Figure S2A,B).

### 3.3 Association between serum tumour markers and plasma miR‐371a‐3p levels

There was correlation between miR‐371a‐3p continuous levels and serum AFP and LDH, and between miR‐371a‐3p dichotomized and LDH (Table 3, Figure S3A). There was a moderate correlation between serum tumour markers defined as S‐stage and plasma miR‐371a‐3p (Table 3, Figure S3B) This was consistent both for seminoma patients (correlation coefficient = 0.32, *P *=* *0.02 as continuous variable and 0.46, *P *=* *0.0006 for dichotomized variable) as well as for non‐seminoma patients (correlation coefficient = 0.42, *P *<* *0.00001 as continuous variable and 0.48, *P *<* *0.00001 for dichotomized miR‐371a‐3p).

**Table 3:** Correlation between pretreatment plasma miR‐371a‐3p and serum tumour markers

Variable	miR‐371a‐3p continuous		miR‐371a‐3p dichotomized	
	Pearson correlation	*P*‐value	Pearson correlation	*P*‐value
AFP	0.26	**0.0025**	0.13	0.14
HCG	−0.02	0.78	0.15	0.08
LDH	0.61	**<0.00001**	0.33	**0.0001**
S‐stage	0.41	**<0.00001**	0.42	**<0.00001**

Statistically significant indicated bold.

HCG, human chorionic gonadotropin; AFP, alpha‐fetoprotein; LDH, lactate dehydrogenase.

In the 101 patients with paired samples, plasma miR‐371a‐3p positivity before the treatment was in 53 (52.5%) of patients, while positivity of serum tumour markers (stage S1‐3) was present in 59 (58.4% of patients). After the first cycle of chemotherapy, only four (4.0%) patients were miR‐371a‐3p positive while in 27 (26.7%) patients, serum tumour markers remained elevated (*P *=* *0.00005).

### 3.4 Prognostic value of plasma miR‐371a‐3p

In the median follow‐up time of 20.9 months (range 0.1‐65.1 months), 15 patients (8.3%) experienced disease progression and 12 patients (6.7%) died. Estimated 2‐year PFS and OS were 89.3%, 95% CI (84.3**‐**94.3%) and 92.2%, 95% CI (87.5**‐**97.0%), respectively.

**Figure 2:**
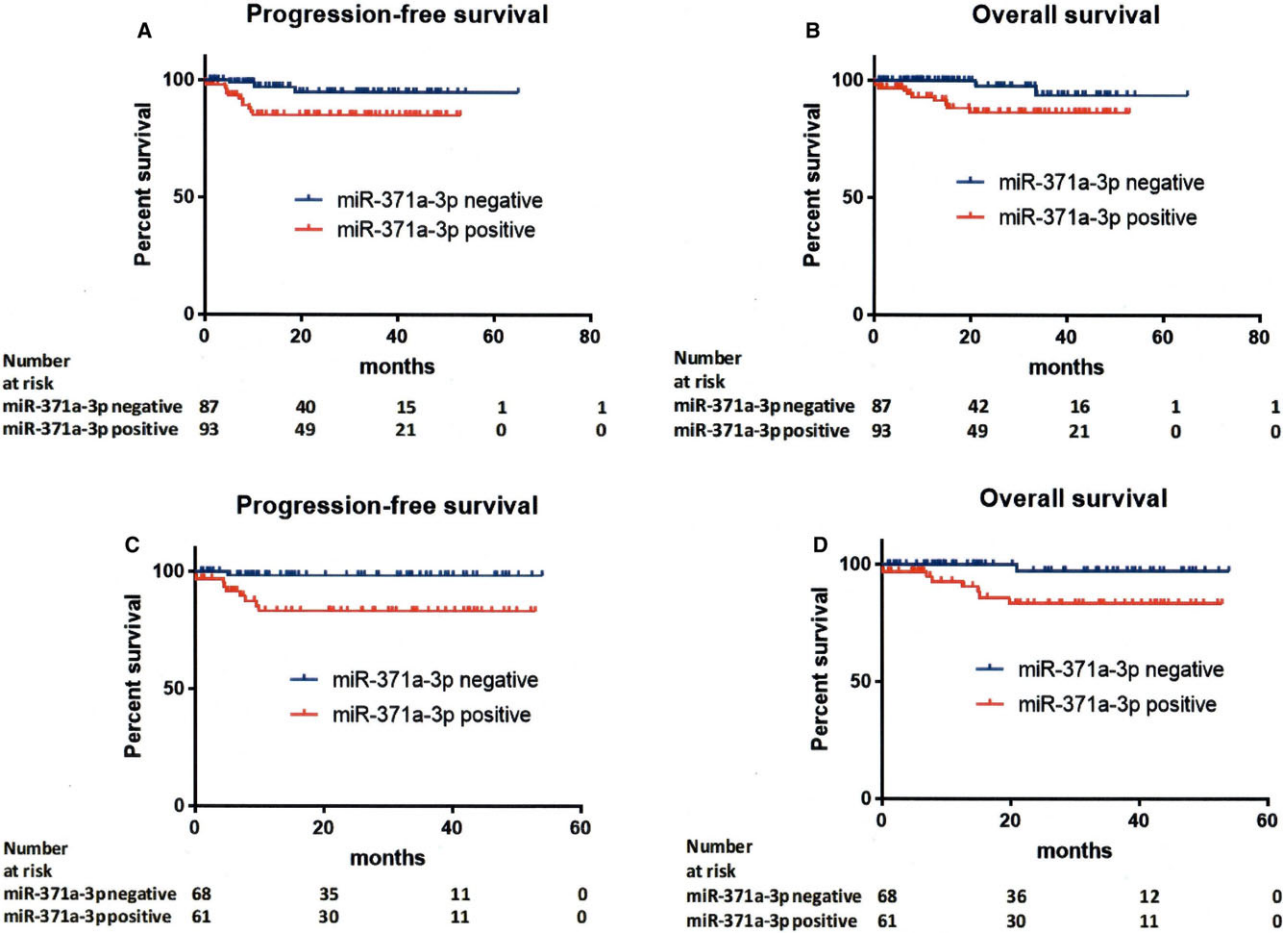
Kaplan‐Meier estimates of probabilities of: (A) progression‐free survival according to relative pretreatment miR‐371a‐3p plasma level in (testicular) germ cell tumour patients (n = 180) (hazard ratio [HR] = 0.26, 95% confidence interval [CI] 0.09‐0.71, *P* = 0.02). Cut‐off for miR‐371a‐3p = 1, based on optimal separation of healthy donors and primary (T)GCT patients was used to dichotomize the miRNA test ampTSmiR; (B) overall survival according to relative pretreatment miR‐371a‐3p plasma level in (testicular) germ cell tumour patients (n = 180) (HR = 0.21, 95% CI 0.07‐0.67, *P* = 0.03); (C) progression‐free survival according to relative pretreatment miR‐371a‐3p plasma level in non‐seminoma patients (n = 129) (HR = 0.10, 95% CI 0.03‐0.33, *P* = 0.006). Cut‐off for miR‐371a‐3p = 1, based on optimal separation of healthy donors and primary (T)GCT patients was used to dichotomize the microRNA‐371a‐3p ampTSmiR test; and (D) overall survival according to relative pretreatment miR‐371a‐3p plasma level in non‐seminoma patients (n = 129) (HR = 0.11, 95% CI 0.03‐0.41, *P* = 0.01)

In patients with negative pretreatment plasma miR‐371a‐3p levels, the biomarker was associated with significantly better PFS and OS compared to patients miR‐371a‐3p positive (hazard ratio [HR] = 0.26, 95% CI 0.09**‐**0.71, *P *=* *0.02 for PFS and HR = 0.21, 95% CI 0.07**‐**0.67, *P *=* *0.03 for OS, respectively) (Table 4, Figure S2A,B). Only three (3.5%) of the patients with negative pretreatment levels of miR‐371a‐3p experienced disease progression and two (2.3%) of the patients died during follow up (median survival 20.9 months). While in seminoma patients the pretreatment plasma levels of miR‐371a‐3p were not prognostic (HR = 1.19, 95% CI 0.19**‐**7.39, *P *=* *0.85 for PFS and HR = 1.04, 95% CI 0.09**‐**11.65, *P *=* *0.98 for OS, respectively) (Figure S4A,B), in non‐seminoma pretreatment plasma levels of miR‐371a‐3p were significantly associated with patients outcome (HR = 0.10, 95% CI 0.03**‐**0.33, *P *=* *0.006 for PFS and HR = 0.11, 95% CI 0.03**‐**0.41, *P *=* *0.01 for OS, respectively) (Figure 2C,D). In subgroups of patients with negative serum tumour markers (S0‐stage) plasma miR‐371a‐3p before the first or the second cycle of chemotherapy was not associated with patients’ outcome, however, only three disease progressions and one death were observed in this subgroup of patients. No progression and/or death was observed in patients with clinical stage IA/B disease. In metastatic tumours (stage IS‐IIIC), negative pretreatment plasma miR‐371a‐3p levels, the biomarker was associated with better PFS and OS compared to patients miR‐371a‐3p positive (HR = 0.39, 95% CI 0.14**‐**1.09, *P *= 0.13 for PFS and HR = 0.34, 95% CI 0.10**‐**1.09, *P *= 0.14 for OS, respectively).

**Table 4:** Prognostic value of plasma miR‐371a‐3p before the first cycle of chemotherapy

Variable	HR (95% CI), *P*‐value			
	Progression‐free survival		Overall survival	
	Univariate analysis	Multivariate analysis	Univariate analysis	Multivariate analysis
Plasma miR‐371a‐3p				
Negative vs. positive	0.26 (0.09‐0.71), **0.02**	0.40 (0.11‐1.47), 0.20	0.21 (0.07‐0.67), **0.03**	0.42 (0.09‐1.98), 0.33
IGCCCG risk group				
Good risk vs. intermediate/poor risk	0.15 (0.05‐0.51), **0.0001**	0.19 (0.06‐0.58), **0.003**	0.07 (0.02‐0.25), **<0.00001**	0.08 (0.020.39), **0.002**

Statistically significant indicated bold.

In multivariate analysis, pretreatment plasma levels of miR‐371a‐3p was not independently associated with IGCCCG risk group for PFS (*P *=* *0.20) or OS (*P *=* *0.33) in the whole population (Table 4) nor in the non‐seminoma subgroup (not shown), while IGCCCG risk group category was associated with patients’ outcome irrespective of plasma miR‐371a‐3p levels.

Plasma miR‐371a‐3p level before the second cycle of chemotherapy (N = 101) was not prognostic for PFS or OS (HR = 0.38, 95% CI 0.02**‐**8.74, *P *=* *0.35 for PFS and HR = 0.30, 95% CI 0.01**‐**9.99, *P *=* *0.22 for OS, respectively) in the whole population nor in the seminoma or non‐seminoma subgroup (data not shown).

In the subgroup of 101 patients with samples available before the first and the second cycle of chemotherapy, patients with a negative miR‐371a‐3p measurement in both samples had a significantly superior PFS (HR = 0.10, 95% CI 0.01**‐**21.49, *P *=* *0.02) and OS (HR = 0.08, 95% CI 0.01**‐**27.81, *P *=* *0.008) compared to patients with miR‐371a‐3p positive in both samples. Patients with at least one positive miR‐371a‐3p sample (baseline, before the second cycle or both) had non‐significantly inferior outcome compared to patients with negative miR‐371a‐3p in both samples (HR = 0.18, 95% CI 0.10**‐**1.34, *P *=* *0.18 for PFS and HR = 0.19, 95% CI 0.10**‐**1.35, *P *=* *0.19 for OS).

## 4 DISCUSSION

In this translational study, we observed a number of associations between several disease characteristics and pretreatment plasma miR‐371a‐3p, including IGCCCG risk group, number of metastatic sites, presence of lung, retroperitoneal and mediastinal lymph node metastases, S – stage, and favourable response to therapy. Moreover, these associations were consistent for plasma levels of miR‐371a‐3p analysed as continuous or dichotomized variable (except for response to therapy).

Plasma miR‐371a‐3p levels were not clearly associated with any histological subtype within the group cohort. This could be due to the low number of more differentiated subtypes like teratoma and or choriocarcinoma and yolk sac tumours. In another study, dysgerminomas possess higher tumour expression of miR‐371a‐3p compared to mixed GCT germ cell tumours that is consistent with our data.^20^ When we analysed stage I patients separately, there were no differences in plasma miR‐371a‐3p levels based on histology of primary tumour. At the time of analysis all but two patients had a persisting primary (T)GCT, 75% of them had metastases while 25% had no remaining tumour. Metastases and primary tumours may have different histologies particularly in those with mixed GCT (the largest group in this cohort). Therefore, the list of histologies is not necessarily identical with the spectrum of histologies actually present at the time of analysis. Therefore, much of the inconsistencies with previous work^10^, ^14^ may come from uncertain histologies. For example, all teratoma patients had elevated serum tumour markers (four patients were S1, one patient S2, and one patient S3) including elevated β‐HCG in three of them suggesting other histologic component in addition to teratoma was present in metastatic sites.

In our study, only 51.7% of the present patients with (T)GCT had elevated levels of miRNA before chemotherapy as opposed to rates around 90% reported in previous studies.^10^, ^14^ This is most likely explained by the fact that in this study the measurements were performed just prior to the start of chemotherapy and not at the moment of orchiectomy. The aim of the study was to show the prognostic value of miR‐371a‐3p in (T)GCTs and not to compare the survival of miR‐371a‐3p positive (T)GCT patients to a healthy population.

Plasma miR‐371a‐3p positivity was found in 13.3% of patients with clinical stage I disease after orchiectomy only. Surprisingly, there was no association between plasma miR‐371a‐3p levels and some prognostic factors associated with poor outcome, like non‐pulmonary visceral metastases or extragonadal GCTs suggesting that miR‐371a‐3p is indeed a marker associated with tumour burden as suggested before,^10^ also in line with the finding that this miR is expressed in all histological elements except teratoma, rather than with treatment resistance. Subgroup analysis revealed that this association is mainly triggered by non‐seminomatous histology. However numerically, in seminoma similar trends were observed as in non‐seminoma.

In our study we observed for the first‐time prognostic value of pretreatment plasma levels of miR‐371a‐3p for PFS and OS in (T)GCTs. Subgroup analysis suggested limited prognostic value in seminoma patients and patients with negative serum tumour markers, e.g. subgroups for whom, the disease response could be evaluated only with imaging studies. However, extremely good prognosis of these subgroups and low number of events preclude to draw any definitive conclusion about the prognostic value of plasma miR‐371a‐3p in these subgroups. Multivariate analysis did not show prognostic value of plasma miR‐371a‐3p levels independently of IGCCCG risk category, an observation that is consistent with strong correlation between several IGCCCG risk factors and plasma miR‐371a‐3p level. Sharp decline in positivity of plasma miR‐371a‐3p after one cycle of cisplatin‐based chemotherapy compared to serum tumour markers suggest rapid clearance of miR‐371a‐3p, in agreement with a recent study^10^ and implies that plasma miR‐371a‐3p changes could be early, very sensitive predictor of treatment effect in TGCTs.^14^, ^21^

Plasma miR‐371a‐3p before the second cycle of chemotherapy showed no prognostic value in the whole patient population, probably due to rapid normalization of plasma miR‐371a‐3p after even one cycle of chemotherapy, an observation consistent with a previous study.^10^ On the other hand, patients with positive miR‐371a‐3p in both samples had inferior outcome compared to continuously negative patients, suggesting that persistence of plasma miR‐371a‐3p positivity could be an early marker of treatment failure in TGCTs. However, due to the low number of patients in subgroups, these results should be confirmed in future studies.

Despite several strengths, this study has some limitations as well, including the retrospective nature of the analysis. Even though this is currently the largest study evaluating plasma miR‐371a‐3p clinical utility in this patient population, low number of events due to very good prognosis of TGCTs preclude from drawing definitive conclusions in some subpopulation of patients. Moreover, due to the low number of events in subgroups for whom the clinical utility of plasma miR‐371a‐3p could be largest due to negativity of serum tumour markers (S0‐stage disease and/or seminoma), we cannot assess the real clinical utility of plasma miR‐371a‐3p in these subgroups. In the study presented here the levels of miR‐371a‐3p have been investigated. We reported an AUC of 0.951, with an 89% specificity and 90% sensitivity for miR‐371a‐3p and an AUC of 0.962 for the combination of the miRs with specificity of 92% and sensitivity of 91%.^14^ However, in our most recent studies addition of the extra miRs compared to miR‐371a‐3p did not improve the outcome.^13^, ^18^ In addition, other studies are based on only using miR‐371.^10^, ^16^, ^21^

In this large translational study we showed for the first time an association between plasma miR‐371a‐3p level and several disease characteristics including sites of metastases, serum tumour markers, IGCCCG prognostic group or favourable response to chemotherapy measured at the time just prior to the start of chemotherapy. Moreover, we observed prognostic value of plasma miR‐371a‐3p in chemotherapy‐naïve GCT patients starting first line of chemotherapy as well as prognostic value of plasma miR‐371a‐3p changes during the treatment suggesting clinical utility of plasma miR‐371a‐3p in TGCTs.

## ACKNOWLEDGEMENTS

We would like to acknowledge Mrs. Daniela Jantekova from the Population Registry of Slovak Republic for help with updating the patient database.

## CONFLICTS OF INTEREST

The authors confirm that there are no conflicts of interest.

## AUTHORS’ CONTRIBUTIONS

M‐M, TvA and L‐L participated in conception and design of this study. M‐M performed the statistical analysis. P‐G processed all blood samples and isolated plasma. TvA performed miRNA analysis in all patient samples and controls. M‐M, M‐CH, V‐M, and J‐M were involved in patient accrual and collection of clinical data. M‐M, TvA, and L‐L drafted the article and all authors reviewed it critically for important intellectual content. All the authors participated in the acquisition, analysis, and interpretation of data, and gave their final approval of the version to be published.
