# Supplementary figures and images for: Clinical utility of plasma miR‐371a‐3p in germ cell tumors
Source: J Cell Mol Med. 2018 Dec 7;23(2):1128–36. doi: 10.1111/jcmm.14013 (PMC6349199; doi:10.1111/jcmm.14013)

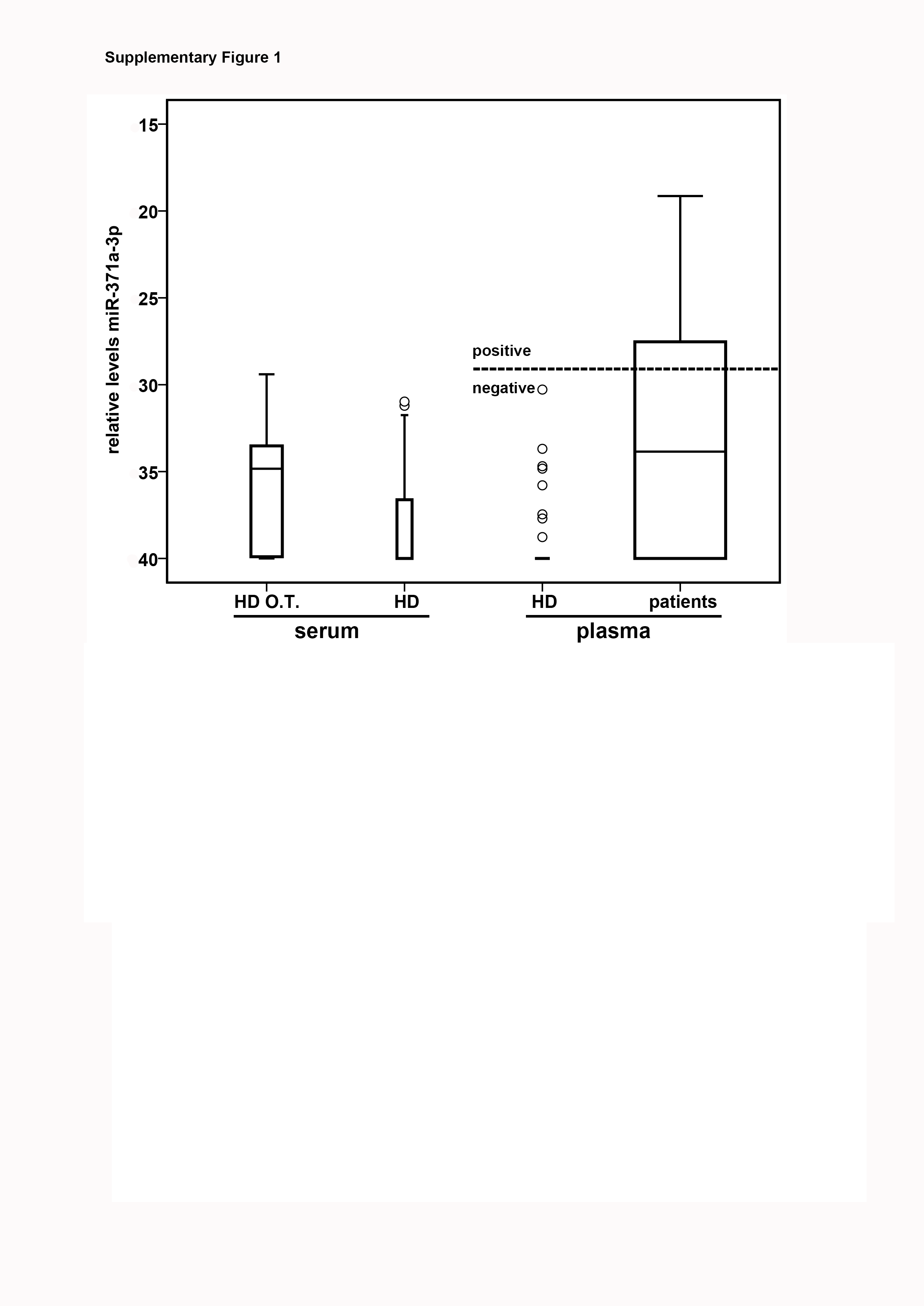

Supplement: Supplementary file 1 [file JCMM-23-1128-s001.tif]

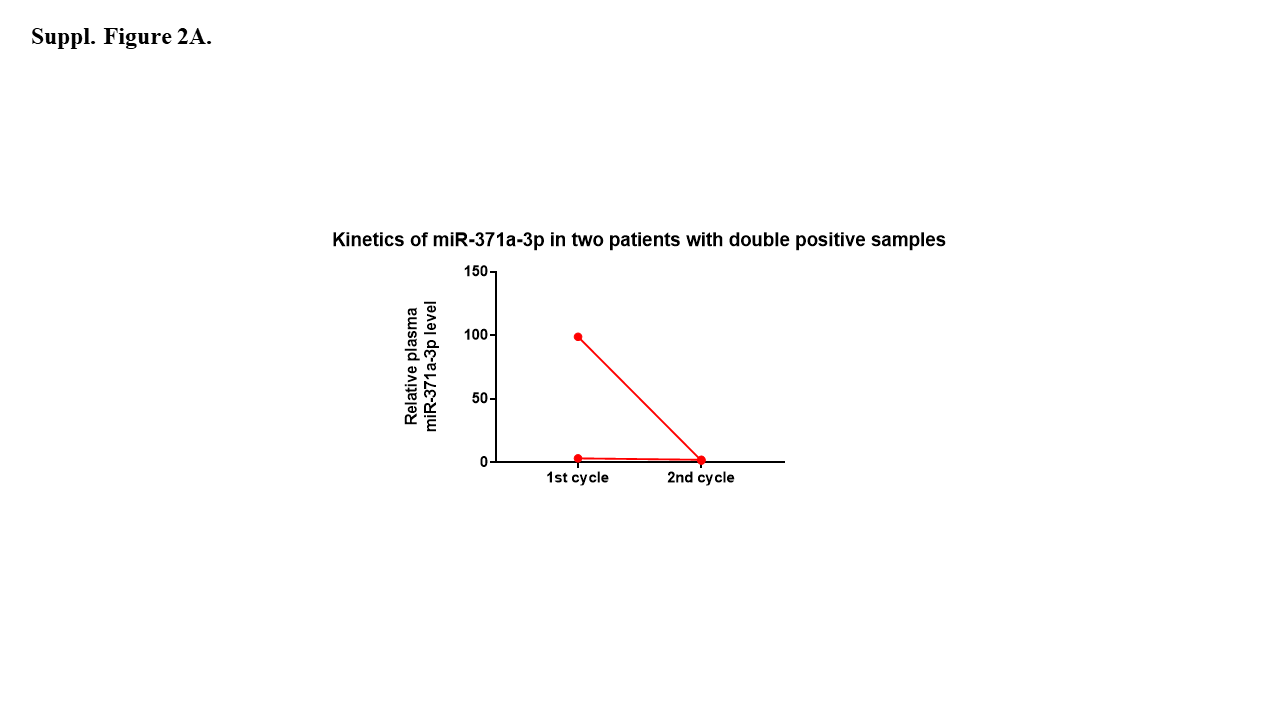

Supplement: Supplementary file 2 [file JCMM-23-1128-s002.TIF]

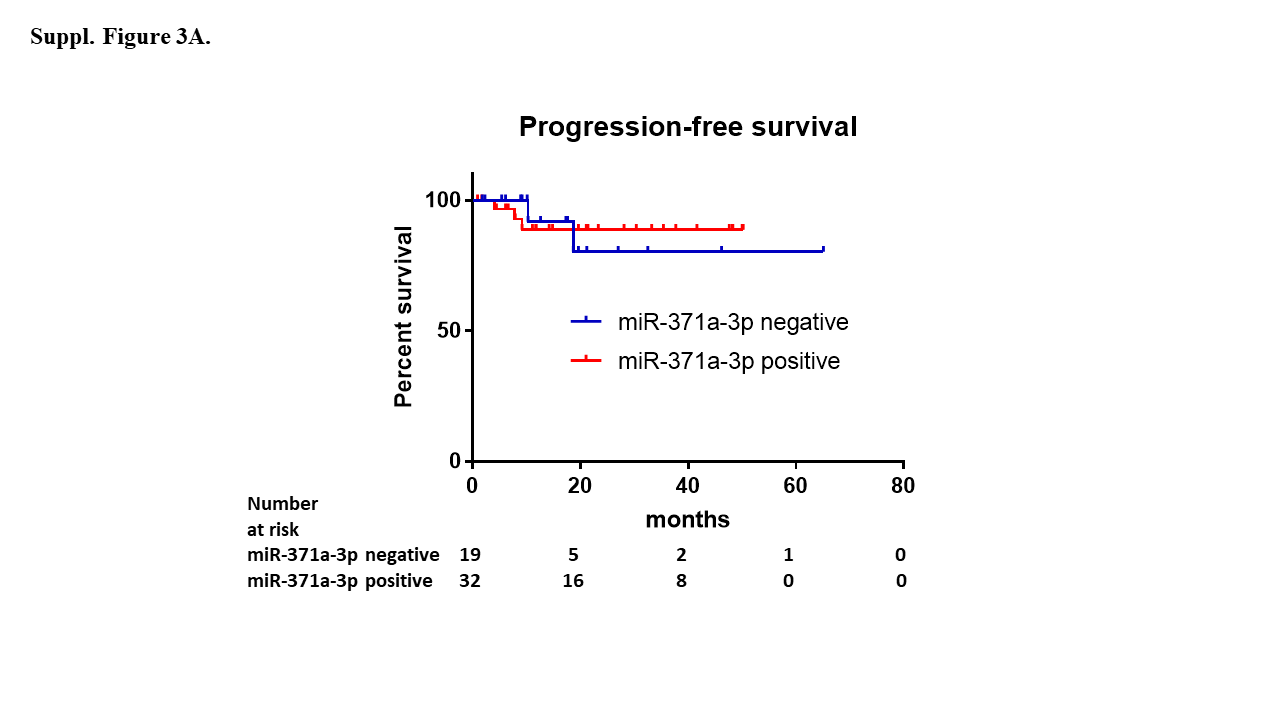

Supplement: Supplementary file 3 [file JCMM-23-1128-s003.TIF]

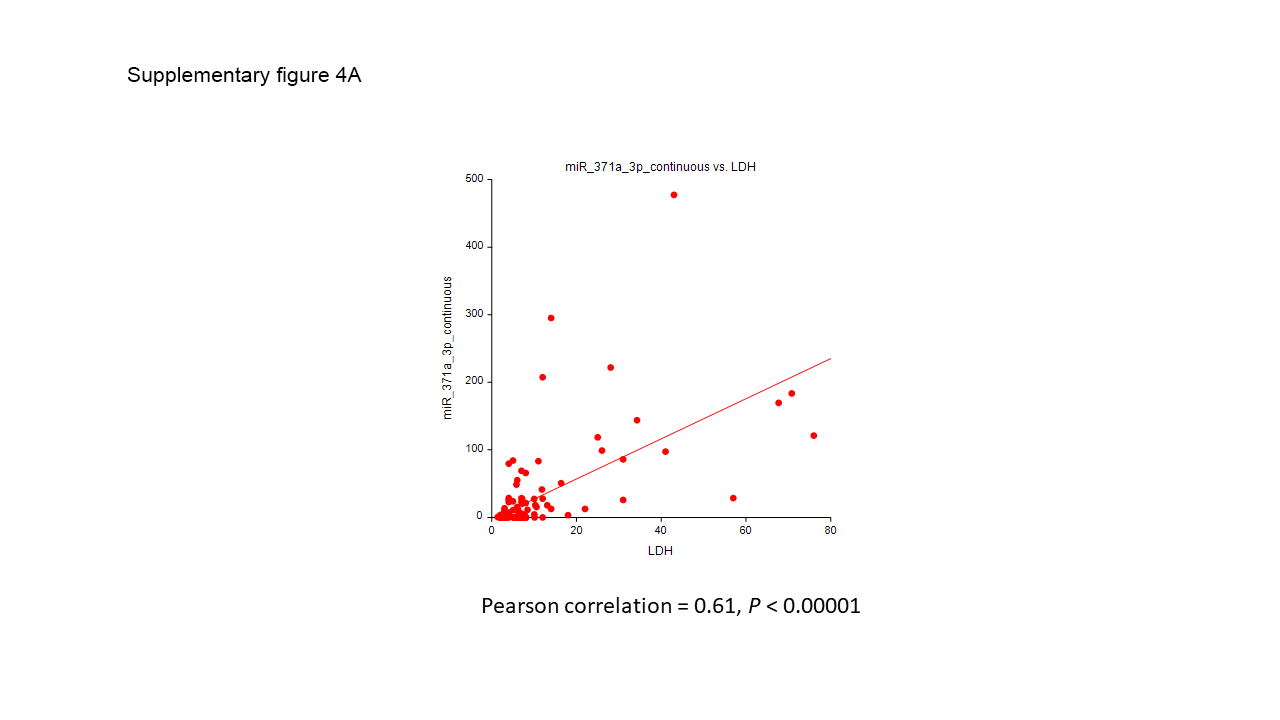

Supplement: Supplementary file 4 [file JCMM-23-1128-s004.tif]
