# Supplementary material for: Clinical utility of plasma miR‐371a‐3p in germ cell tumors
Source: J Cell Mol Med. 2018 Dec 7;23(2):1128–36. doi: 10.1111/jcmm.14013 (PMC6349199; doi:10.1111/jcmm.14013)
Supplement: Supplementary file 5 [file JCMM-23-1128-s005.docx]

**Supplementary material**

**Table S1.** **Association between miR-371a-3p and patients/tumor characteristics – seminoma**

| **Variable** | **N** | **Mean** | **Median** | **SD** | **SEM** | ***P*-value** |
| --- | --- | --- | --- | --- | --- | --- |
| **Seminoma** | 51 | 36.6 | 3.4 | 80.5 | 11.3 | NA |
| **Tumor primary** |  |  |  |  |  |  |
| Primary TGCTs | 46 | 40.4 | 4.3 | 83.9 | 11.9 | 0.14 |
| Extragonadal GCTs | 5 | 2.0 | 0.2 | 4.2 | 36.0 |  |
| **IGCCCG risk group** |  |  |  |  |  |  |
| Good risk | 44 | 32.4 | 3.3 | 81.6 | 12.2 | 0.51 |
| Intermediate risk | 7 | 80.4 | 97.4 | 79.8 | 36.1 |  |
| Stage I (adjuvatnt therapy) | 0 | NA | NA | NA | NA |  |
| **Number of metastatic sites** |  |  |  |  |  |  |
| 0 | 0 | NA | NA | NA | NA | 0.10 |
| 1 to 2 | 46 | 26.9 | 2.6 | 57.0 | 11.1 |  |
| > 3 | 5 | 126.1 | 39.9 | 182.4 | 33.8 |  |
| **Retroperitoneal lymph nodes metastases** |  |  |  |  |  |  |
| Absent | 7 | 34.4 | 0.2 | 58.3 | 30.7 | 0.60 |
| Present | 44 | 37.0 | 4.3 | 84.0 | 12.3 |  |
| **Mediastinal lymph nodes metastases** |  |  |  |  |  |  |
| Absent | 45 | 25.8 | 3.2 | 55.8 | 11.2 | 0.11 |
| Present | 6 | 117.6 | 62.9 | 168.5 | 30.8 |  |
| **Lung metastases** |  |  |  |  |  |  |
| Absent | 48 | 33.3 | 2.6 | 80.3 | 11.6 | 0.08 |
| Present | 3 | 89.4 | 121.1 | 75.4 | 46.3 |  |
| **Liver metastases** |  |  |  |  |  |  |
| Absent | 49 | 37.3 | 3.4 | 81.9 | 11.6 | 0.84 |
| Present | 2 | 19.9 | 19.9 | 28.2 | 57.4 |  |
| **Non-pulmonary visceral metastases** |  |  |  |  |  |  |
| Absent | 47 | 38.9 | 3.8 | 83.3 | 11.8 | 0.27 |
| Present | 4 | 10.0 | 0.1 | 19.9 | 40.4 |  |
| **S – stage** |  |  |  |  |  |  |
| 0 | 27 | 3.2 | 0.0 | 6.1 | 13.7 | 0.00016 |
| 1 | 14 | 51.9 | 10.2 | 114.1 | 19.1 |  |
| 2 | 5 | 122.7 | 118.6 | 110.8 | 31.9 |  |
| 3 | 5 | 88.4 | 97.4 | 71.4 | 31.9 |  |
| **Response to therapy** |  |  |  |  |  |  |
| Favourable response | 50 | 33.7 | 3.3 | 78.5 | 11.1 | 0.11 |
| Unfavourable response | 1 | 183.5 | 183.5 |  | 78.5 |  |

**Table S2.** **Association between miR-371a-3p and patients/tumor characteristics – non-seminoma**

| **Variable** | **N** | **Mean** | **Median** | **SD** | **SEM** | ***P*-value** |
| --- | --- | --- | --- | --- | --- | --- |
| **Non-seminoma** | 129 | 21.1 | 0.9 | 58.0 | 5.1 | NA |
| **Tumor primary** |  |  |  |  |  |  |
| Primary TGCTs | 123 | 19.5 | 0.6 | 57.1 | 5.2 | **0.03** |
| Extragonadal GCTs | 6 | 54.7 | 19.1 | 72.3 | 23.6 |  |
| **IGCCCG risk group** |  |  |  |  |  |  |
| Good risk | 61 | 8.8 | 0.9 | 16.3 | 6.8 | **< 0.00001** |
| Intermediate risk | 16 | 49.7 | 21.6 | 65.4 | 13.3 |  |
| Poor risk | 20 | 67.6 | 14.7 | 119.6 | 11.9 |  |
| Stage I (adjuvatnt therapy) | 32 | 1.2 | 0.0 | 4.7 | 9.4 |  |
| **Number of metastatic sites** |  |  |  |  |  |  |
| 0 | 45 | 1.1 | 0.0 | 4.0 | 8.0 | **< 0.00001** |
| 1 to 2 | 63 | 19.0 | 3.6 | 38.4 | 6.8 |  |
| > 3 | 21 | 70.2 | 27.8 | 116.1 | 11.7 |  |
| **Retroperitoneal lymph nodes metastases** |  |  |  |  |  |  |
| Absent | 50 | 5.4 | 0.0 | 31.5 | 8.0 | **< 0.00001** |
| Present | 79 | 31.0 | 10.7 | 68.1 | 6.4 |  |
| **Mediastinal lymph nodes metastases** |  |  |  |  |  |  |
| Absent | 114 | 12.4 | 0.3 | 31.0 | 5.0 | **0.00027** |
| Present | 15 | 87.2 | 28.5 | 133.0 | 13.7 |  |
| **Lung metastases** |  |  |  |  |  |  |
| Absent | 96 | 11.0 | 0.2 | 34.7 | 5.7 | **0.00001** |
| Present | 33 | 50.3 | 21.3 | 93.3 | 9.7 |  |
| **Liver metastases** |  |  |  |  |  |  |
| Absent | 120 | 17.5 | 0.6 | 42.7 | 5.2 | 0.10 |
| Present | 9 | 69.0 | 12.6 | 154.5 | 18.9 |  |
| **Non-pulmonary visceral metastases** |  |  |  |  |  |  |
| Absent | 119 | 17.6 | 0.5 | 42.9 | 5.2 | 0.12 |
| Present | 10 | 62.2 | 10.2 | 147.3 | 18.0 |  |
| **S – stage** |  |  |  |  |  |  |
| 0 | 46 | 1.8 | 0.0 | 5.3 | 7.8 | **< 0.00001** |
| 1 | 47 | 10.5 | 1.3 | 17.9 | 7.7 |  |
| 2 | 22 | 47.2 | 19.3 | 64.6 | 11.2 |  |
| 3 | 14 | 78,9 | 17,4 | 136,7 | 14,1 |  |
| **Response to therapy** |  |  |  |  |  |  |
| Favourable response | 123 | 19.6 | 0.5 | 56.5 | 5.2 | **0.04** |
| Unfavourable response | 6 | 51.0 | 10.2 | 84.3 | 23.6 |  |

**Table S3.** **Association between miR-371a-3p and patients/tumor characteristics – S0 stage patients**

| **Variable** | **N** | **Mean** | **Median** | **SD** | **SEM** | **p-value** |
| --- | --- | --- | --- | --- | --- | --- |
|  |  |  |  |  |  |  |
| **S0 stage patients** | 73 | 2.3 | 0.0 | 5.6 | 0.7 | NA |
| **Histology** |  |  |  |  |  |  |
| Seminoma | 27 | 3.2 | 0.0 | 6.1 | 1.1 | 0.43 |
| Non-seminoma | 46 | 1.8 | 0.0 | 5.3 | 0.8 |  |
| **Tumor primary** |  |  |  |  |  |  |
| Primary TGCTs | 69 | 2.3 | 0.0 | 5.7 | 0.7 | 0.98 |
| Extragonadal GCTs | 4 | 2.4 | 0.1 | 4.7 | 2.8 |  |
| **IGCCCG risk group** |  |  |  |  |  |  |
| Good prognosis | 40 | 3.2 | 0.0 | 6.2 | 0.9 | 0.27 |
| Intermediate | 1 | 0.0 | 0.0 | NA | 5.6 |  |
| Poor prognosis | 0 | NA | NA | NA | NA |  |
| Stage I (adjuvatnt therapy) | 32 | 1.2 | 0.0 | 4.7 | 1.0 |  |
| **Number of metastatic sites** |  |  |  |  |  |  |
| 0 | 32 | 1.2 | 0.0 | 4.7 | 1.0 | 0.24 |
| 1 to 2 | 41 | 3.2 | 0.0 | 6.1 | 0.9 |  |
| > 3 | 0 | NA | NA | NA | NA |  |
| **Retroperitoneal lymph nodes metastases** |  |  |  |  |  |  |
| Absent | 37 | 1.1 | 0.0 | 4.3 | 0.9 | 0.11 |
| Present | 36 | 3.6 | 0.0 | 6.4 | 0.9 |  |
| **Mediastinal lymph nodes metastases** |  |  |  |  |  |  |
| Absent | 71 | 2.3 | 0.0 | 5.6 | 0.7 | 0.24 |
| Present | 2 | 3.6 | 3.6 | 5.1 | 4.0 |  |
| **Lung metastases** |  |  |  |  |  |  |
| Absent | 70 | 2.0 | 0.0 | 5.2 | 0.7 | 0.27 |
| Present | 3 | 8.2 | 3.4 | 11.4 | 3.2 |  |
| **Liver** |  |  |  |  |  |  |
| Absent | 73 | 2.3 | 0.0 | 5.6 | 0.7 | NA |
| Present | 0 | NA | NA | NA | NA |  |
| **Non-pulmonary visceral metastases** |  |  |  |  |  |  |
| Absent | 72 | 2.3 | 0.0 | 5.6 | 0.7 | 0.32 |
| Present | 1 | 0.0 | 0.0 | NA | 5.6 |  |
| **Response to therapy** |  |  |  |  |  |  |
| Favourable response | 73 | 2.3 | 0.0 | 5.6 | 0.7 | NA |
| Unfavourable response | 0 | NA | NA | NA | NA |  |

**Table S4. The associations between the histological subtypes of the primary germ cell tumors and the** **pre-treatment plasma miR-371a-3p levels**

| **Histological subtype *** | **N** | **Mean** | **SEM** | **Median** | **P-value**** |
| --- | --- | --- | --- | --- | --- |
| Seminoma | 51 | 35.9 | 11.1 | 3.0 | NA |
| Embryonal carcnoma | 24 | 17.9 | 9.4 | 0.5 | 0.28 |
| Yolk sac tumors | 7 | 62.2 | 36.6 | 7.9 | 0.39 |
| Choriocarcinoma | 6 | 11.9 | 3.8 | 12.6 | 0.50 |
| Teratoma | 6 | 54.0 | 29.1 | 15.5 | 0.10 |
| Mixed germ cell tumors | 84 | 16.6 | 6.3 | 0.3 | 0.08 |

* In 2 patients, chemotherapy started without histological confirmation due to very advanced disease.

** compared to seminoma

Abbreviations: SEM, standard error of the mean
